# Tri-μ-ethanethiol­ato-bis­{[η^5^-1,2,3,4-tetra­methyl-5-(trimethyl­silyl)cyclo­penta­dien­yl]iron(II,III)}(*Fe^II^*–*Fe^III^*)

**DOI:** 10.1107/S1600536809045735

**Published:** 2009-11-07

**Authors:** Jing Li

**Affiliations:** a Key Laboratory of Food Nutrition and Safety, Ministry of Education, College of Food Engineering and Biotechnology, Tianjin University of Science and Technology, Tianjin 300457, People’s Republic of China

## Abstract

The title complex, [Fe_2_(C_2_H_5_S)_3_(C_12_H_21_Si)_2_], has an unusual Fe_2_S_3_ core. The two 1,2,3,4-tetra­methyl-5-(trimethyl­silyl)cyclo­penta­dienyl (Cp′) ligands coordinate to the Fe atoms with their C_5_ planes perpendicular [dihedral angles = 88.23 (7) and 88.55 (7)°] to the Fe—Fe vector, building two Cp′Fe subunits. These two subunits are bridged by three thiol­ate ligands. There are no significant differences in the coordination geometries between the two Fe atoms. The short Fe—Fe distance of 2.7842 (5) Å is clear evidence of an inter­metallic bond. Such a diiron–sulfur structure might act as a model of active sites in some metalloproteins.

## Related literature

For related diiron clusters, [CpFe(μ-S*R*)_3_FeCp^*^] (Cp = η^5^-C_5_Me_5_, *R* = Me, Et and Ph) and [CpFe(μ-SMe)_3_FeCp], see: Chen *et al.* (2008*a* ▶,*b* ▶); Madec *et al.* (1999 ▶).
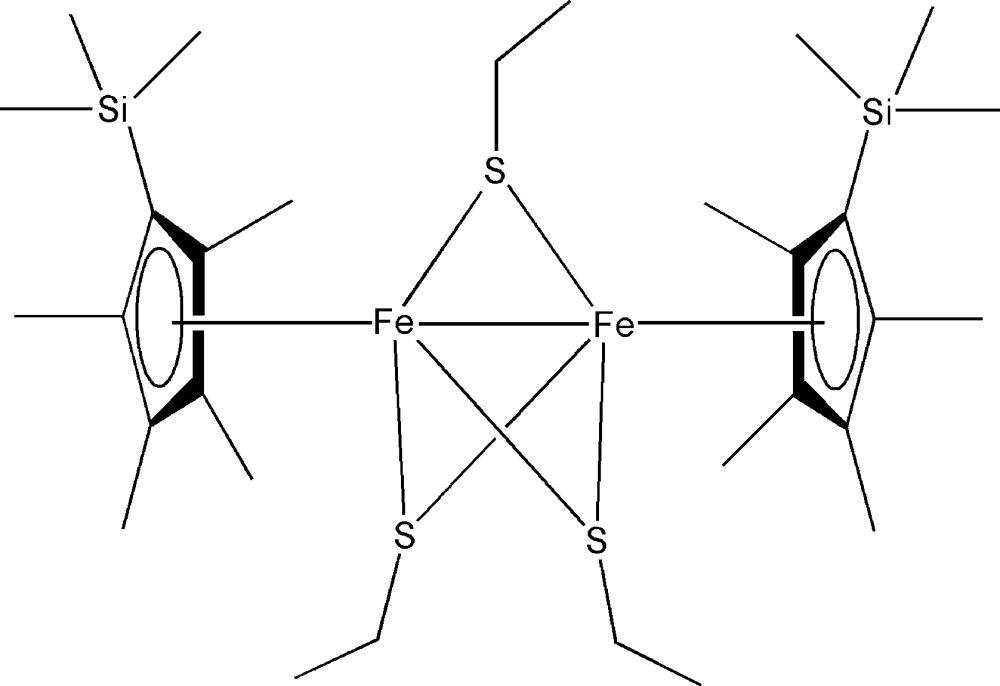


## Experimental

### 

#### Crystal data

[Fe_2_(C_2_H_5_S)_3_(C_12_H_21_Si)_2_]
*M* *_r_* = 681.82
Orthorhombic, 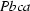

*a* = 17.7426 (19) Å
*b* = 19.874 (2) Å
*c* = 20.493 (2) Å
*V* = 7226.2 (13) Å^3^
*Z* = 8
Mo *K*α radiation
μ = 1.06 mm^−1^
*T* = 293 K
0.55 × 0.43 × 0.21 mm

#### Data collection

Bruker SMART APEX CCD diffractometer
Absorption correction: multi-scan (*SADABS*; Sheldrick, 1996 ▶) *T*_min_ = 0.593, *T*_max_ = 0.808
43018 measured reflections
6541 independent reflections
4961 reflections with *I* > 2σ(*I*)
*R*_int_ = 0.055

#### Refinement

*R*[*F* ^2^ > 2σ(*F* ^2^)] = 0.034
*wR*(*F* ^2^) = 0.095
*S* = 1.02
6541 reflections
334 parameters
H-atom parameters constrained
Δρ_max_ = 0.45 e Å^−3^
Δρ_min_ = −0.25 e Å^−3^

### 

Data collection: *SMART* (Bruker, 2007 ▶); cell refinement: *SAINT* (Bruker, 2007 ▶); data reduction: *SAINT*; program(s) used to solve structure: *SHELXTL* (Sheldrick, 2008 ▶); program(s) used to refine structure: *SHELXTL*; molecular graphics: *SHELXTL*; software used to prepare material for publication: *SHELXTL*.

## Supplementary Material

Crystal structure: contains datablocks I, global. DOI: 10.1107/S1600536809045735/hy2242sup1.cif

Structure factors: contains datablocks I. DOI: 10.1107/S1600536809045735/hy2242Isup2.hkl

Additional supplementary materials:  crystallographic information; 3D view; checkCIF report

## Figures and Tables

**Table 1:** Selected bond lengths (Å)

Fe1—C13	2.104 (2)
Fe1—C14	2.111 (2)
Fe1—C15	2.126 (3)
Fe1—C16	2.136 (3)
Fe1—C17	2.123 (2)
Fe1—S1	2.2721 (7)
Fe1—S2	2.2765 (7)
Fe1—S3	2.2522 (7)
Fe2—C1	2.109 (2)
Fe2—C2	2.113 (2)
Fe2—C3	2.125 (2)
Fe2—C4	2.135 (2)
Fe2—C5	2.126 (2)
Fe2—S1	2.2659 (7)
Fe2—S2	2.2723 (7)
Fe2—S3	2.2545 (7)
Fe1—Fe2	2.7842 (5)

**Fig. 1.**
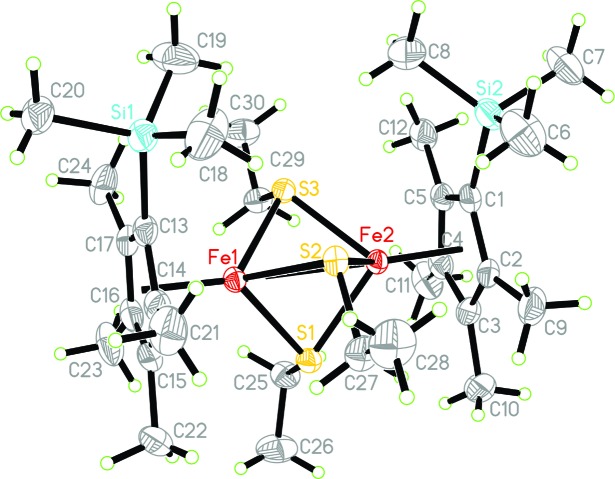
The molecular structure of the title compound, showing displacement ellipsoids at the 30% probability level.

**Fig. 2.**
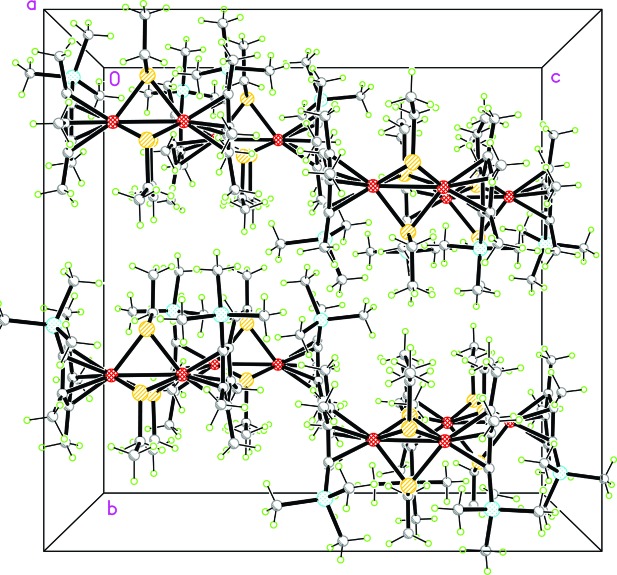
Packing diagram viewed along the a axis.

## supplementary crystallographic information

### Comment

As shown in Fig. 1, the title compound is a dimeric complex, in which each Fe atom is coordinated by a 1,2,3,4-tetramethyl-5-(trimethylsilyl)cyclopentadienyl (Cp') ligand and three ethanethiolate ligands. The C_5_ planes of the two Cp' ligands are perpendicular to the Fe—Fe vector, with angles of 1.89 (7) and 1.45 (7)° between the normals of the planes and the vector. Three thiolate ligands bridge two Fe atoms (Table 1). The plane of the three S atoms is approximately parallel to the Cp' planes with dihedral angles of 1.77 (8) and 1.55 (8)°, respectively, and bisects the Fe—Fe bond. There are no significant differences in the coordination geometries between the two Fe centers. The short Fe—Fe distance of 2.7842 (5) Å is clear evidence of intermetallic bond.

### Experimental

To a stirred suspension of Cp'Li (1.28 g, 6.38 mmol) in 50 ml THF was added anhydrous FeCl_2_ (0.81 g, 6.38 mmol) at 0°C, followed by stirring for 1 h. The resultant olive-green [Cp'FeCl]_2_ solution was cooled to -78°C. Then, a suspension of LiSEt in THF, which was prepared by reaction of *n*-BuLi (2.20 ml, 2.9 *M* solution in *n*-hexane) and HSEt (0.48 ml, 6.38 mmol) at 0°C, was transferred *via* a cannula to the cooled solution of [Cp'FeCl]_2_. The mixture was placed in a -78°C bath for 1 h and stirred overnight as it warmed to ambient temperature. The resulting red-violet solution was evaporated to dryness, and the residue was purified by column chromatography on neutral alumina with *n*-hexane as the eluent to give complex [Cp'Fe(µ-SEt)_3_FeCp'] (yield 0.42 g, 19%) as violet microcrystalline solid. The crystals of the title complex suitable for X-ray analysis were obtained from a benzene solution layered with acetonitrile.

### Refinement

H atoms were visible in difference Fourier maps and were subsequently treated as riding atoms, with C—H = 0.96 (CH_3_) and 0.97 (CH_2_) Å and with *U*_iso_(H) = 1.2(1.5 for methyl)*U*_eq_(C).

### Crystal data

**Table:**

[Fe_2_(C_2_H_5_S)_3_(C_12_H_21_Si)_2_]	*F*(000) = 2920
*M**_r_* = 681.82	*D*_x_ = 1.253 Mg m^−^^3^
Orthorhombic, *P**b**c**a*	Mo *K*α radiation, λ = 0.71073 Å
Hall symbol: -P 2ac 2ab	Cell parameters from 6361 reflections
*a* = 17.7426 (19) Å	θ = 2.3–25.3°
*b* = 19.874 (2) Å	µ = 1.06 mm^−^^1^
*c* = 20.493 (2) Å	*T* = 293 K
*V* = 7226.2 (13) Å^3^	Prism, violet-red
*Z* = 8	0.55 × 0.43 × 0.21 mm

### Data collection

**Table:**

Bruker SMART APEX CCD diffractometer	6541 independent reflections
Radiation source: fine-focus sealed tube	4961 reflections with *I* > 2σ(*I*)
graphite	*R*_int_ = 0.055
φ and ω scans	θ_max_ = 25.3°, θ_min_ = 2.3°
Absorption correction: multi-scan (*SADABS*; Sheldrick, 1996)	*h* = −21→21
*T*_min_ = 0.593, *T*_max_ = 0.808	*k* = −23→23
43018 measured reflections	*l* = −24→24

### Refinement

**Table:**

Refinement on *F*^2^	Primary atom site location: structure-invariant direct methods
Least-squares matrix: full	Secondary atom site location: difference Fourier map
*R*[*F*^2^ > 2σ(*F*^2^)] = 0.034	Hydrogen site location: inferred from neighbouring sites
*wR*(*F*^2^) = 0.095	H-atom parameters constrained
*S* = 1.02	*w* = 1/[σ^2^(*F*_o_^2^) + (0.0482*P*)^2^ + 2.1955*P*] where *P* = (*F*_o_^2^ + 2*F*_c_^2^)/3
6541 reflections	(Δ/σ)_max_ = 0.001
334 parameters	Δρ_max_ = 0.45 e Å^−^^3^
0 restraints	Δρ_min_ = −0.25 e Å^−^^3^

### Fractional atomic coordinates and isotropic or equivalent isotropic displacement parameters (Å^2^)

**Table:**

	*x*	*y*	*z*	*U*_iso_*/*U*_eq_	
Fe1	0.736349 (19)	0.186763 (16)	0.096497 (15)	0.03463 (10)	
Fe2	0.728449 (18)	0.184706 (16)	0.232172 (15)	0.03427 (10)	
S1	0.82127 (3)	0.22827 (3)	0.16907 (3)	0.04064 (15)	
S2	0.74171 (4)	0.09562 (3)	0.16363 (3)	0.04026 (15)	
S3	0.63846 (3)	0.21575 (3)	0.16057 (3)	0.03643 (14)	
Si1	0.60803 (5)	0.08543 (4)	0.00400 (4)	0.0579 (2)	
Si2	0.60737 (5)	0.06522 (4)	0.31233 (4)	0.0592 (2)	
C1	0.67711 (15)	0.13606 (13)	0.31210 (11)	0.0447 (6)	
C2	0.75840 (15)	0.13379 (14)	0.31891 (12)	0.0473 (6)	
C3	0.78633 (16)	0.20045 (16)	0.32168 (12)	0.0523 (7)	
C4	0.72446 (16)	0.24564 (14)	0.31789 (12)	0.0493 (6)	
C5	0.65749 (15)	0.20626 (13)	0.31313 (11)	0.0457 (6)	
C6	0.6515 (3)	−0.01943 (17)	0.3035 (2)	0.0983 (13)	
H6A	0.6130	−0.0533	0.3040	0.147*	
H6B	0.6857	−0.0270	0.3391	0.147*	
H6C	0.6786	−0.0215	0.2630	0.147*	
C7	0.5588 (2)	0.0664 (2)	0.39283 (18)	0.0925 (12)	
H7A	0.5226	0.0306	0.3946	0.139*	
H7B	0.5336	0.1087	0.3985	0.139*	
H7C	0.5952	0.0605	0.4270	0.139*	
C8	0.5375 (2)	0.0728 (2)	0.24510 (19)	0.0990 (13)	
H8A	0.5030	0.0356	0.2470	0.149*	
H8B	0.5633	0.0724	0.2039	0.149*	
H8C	0.5102	0.1142	0.2497	0.149*	
C9	0.8061 (2)	0.07234 (18)	0.33032 (15)	0.0738 (10)	
H9A	0.8083	0.0629	0.3762	0.111*	
H9B	0.8562	0.0803	0.3142	0.111*	
H9C	0.7845	0.0346	0.3078	0.111*	
C10	0.86772 (19)	0.2199 (2)	0.33088 (15)	0.0799 (11)	
H10A	0.8786	0.2235	0.3766	0.120*	
H10B	0.8769	0.2625	0.3102	0.120*	
H10C	0.8995	0.1862	0.3117	0.120*	
C11	0.7284 (2)	0.32007 (16)	0.33017 (15)	0.0752 (11)	
H11A	0.7251	0.3285	0.3762	0.113*	
H11B	0.6873	0.3420	0.3083	0.113*	
H11C	0.7753	0.3373	0.3138	0.113*	
C12	0.57926 (17)	0.23407 (16)	0.31318 (14)	0.0616 (8)	
H12A	0.5617	0.2381	0.3573	0.092*	
H12B	0.5466	0.2044	0.2894	0.092*	
H12C	0.5792	0.2776	0.2929	0.092*	
C13	0.68795 (15)	0.14699 (12)	0.01108 (11)	0.0445 (6)	
C14	0.76750 (16)	0.13311 (15)	0.01178 (12)	0.0513 (7)	
C15	0.80807 (16)	0.19429 (16)	0.01372 (12)	0.0558 (7)	
C16	0.75472 (18)	0.24840 (15)	0.01263 (12)	0.0540 (7)	
C17	0.68159 (16)	0.21948 (13)	0.01000 (11)	0.0472 (6)	
C18	0.6325 (3)	0.00125 (19)	0.0373 (2)	0.1132 (17)	
H18A	0.6769	−0.0153	0.0159	0.170*	
H18B	0.5915	−0.0293	0.0297	0.170*	
H18C	0.6417	0.0048	0.0833	0.170*	
C19	0.5210 (2)	0.1144 (2)	0.0469 (2)	0.1054 (15)	
H19A	0.4820	0.0813	0.0418	0.158*	
H19B	0.5046	0.1563	0.0285	0.158*	
H19C	0.5317	0.1205	0.0925	0.158*	
C20	0.58613 (19)	0.07463 (16)	−0.08434 (14)	0.0691 (9)	
H20A	0.6303	0.0592	−0.1068	0.104*	
H20B	0.5704	0.1169	−0.1024	0.104*	
H20C	0.5464	0.0422	−0.0893	0.104*	
C21	0.8032 (2)	0.06465 (18)	0.00316 (16)	0.0859 (11)	
H21A	0.8098	0.0556	−0.0425	0.129*	
H21B	0.7711	0.0309	0.0220	0.129*	
H21C	0.8513	0.0639	0.0245	0.129*	
C22	0.89283 (18)	0.2019 (2)	0.01279 (16)	0.0888 (12)	
H22A	0.9101	0.2038	−0.0316	0.133*	
H22B	0.9155	0.1641	0.0344	0.133*	
H22C	0.9067	0.2426	0.0350	0.133*	
C23	0.7726 (2)	0.32161 (17)	0.00420 (16)	0.0849 (12)	
H23A	0.7774	0.3317	−0.0414	0.127*	
H23B	0.8191	0.3318	0.0260	0.127*	
H23C	0.7328	0.3482	0.0226	0.127*	
C24	0.61042 (19)	0.25890 (17)	0.00099 (15)	0.0740 (9)	
H24A	0.6011	0.2652	−0.0448	0.111*	
H24B	0.6154	0.3019	0.0218	0.111*	
H24C	0.5691	0.2348	0.0202	0.111*	
C25	0.82319 (17)	0.32059 (13)	0.17175 (14)	0.0532 (7)	
H25A	0.7906	0.3383	0.1379	0.064*	
H25B	0.8039	0.3358	0.2135	0.064*	
C26	0.90144 (19)	0.34718 (17)	0.16224 (19)	0.0828 (11)	
H26A	0.9006	0.3954	0.1639	0.124*	
H26B	0.9203	0.3328	0.1206	0.124*	
H26C	0.9336	0.3303	0.1962	0.124*	
C27	0.83986 (17)	0.06546 (14)	0.16658 (14)	0.0586 (7)	
H27A	0.8670	0.0821	0.1288	0.070*	
H27B	0.8643	0.0831	0.2053	0.070*	
C28	0.8435 (2)	−0.01107 (17)	0.1676 (2)	0.0931 (13)	
H28A	0.8953	−0.0252	0.1692	0.140*	
H28B	0.8202	−0.0286	0.1290	0.140*	
H28C	0.8175	−0.0276	0.2054	0.140*	
C29	0.62541 (15)	0.30762 (12)	0.16122 (13)	0.0470 (6)	
H29A	0.6473	0.3261	0.2007	0.056*	
H29B	0.6515	0.3272	0.1242	0.056*	
C30	0.54183 (17)	0.32631 (15)	0.15798 (17)	0.0676 (9)	
H30A	0.5367	0.3744	0.1584	0.101*	
H30B	0.5161	0.3076	0.1950	0.101*	
H30C	0.5203	0.3086	0.1186	0.101*	

### Atomic displacement parameters (Å^2^)

**Table:**

	*U*^11^	*U*^22^	*U*^33^	*U*^12^	*U*^13^	*U*^23^
Fe1	0.0387 (2)	0.03572 (19)	0.02946 (18)	−0.00254 (14)	0.00126 (13)	−0.00052 (13)
Fe2	0.0365 (2)	0.03658 (19)	0.02974 (18)	−0.00070 (14)	−0.00060 (13)	−0.00037 (13)
S1	0.0380 (3)	0.0448 (3)	0.0391 (3)	−0.0054 (3)	0.0007 (3)	−0.0013 (2)
S2	0.0494 (4)	0.0343 (3)	0.0371 (3)	0.0012 (3)	−0.0001 (3)	−0.0009 (2)
S3	0.0367 (3)	0.0374 (3)	0.0351 (3)	0.0004 (2)	−0.0010 (2)	0.0004 (2)
Si1	0.0693 (5)	0.0598 (5)	0.0447 (4)	−0.0209 (4)	−0.0060 (4)	−0.0041 (3)
Si2	0.0644 (5)	0.0614 (5)	0.0518 (5)	−0.0157 (4)	0.0047 (4)	0.0094 (4)
C1	0.0519 (16)	0.0523 (15)	0.0298 (12)	−0.0016 (12)	0.0021 (11)	0.0045 (10)
C2	0.0490 (15)	0.0613 (17)	0.0315 (12)	0.0037 (13)	−0.0039 (11)	0.0073 (11)
C3	0.0518 (16)	0.0732 (19)	0.0318 (13)	−0.0079 (15)	−0.0071 (11)	−0.0038 (12)
C4	0.0638 (18)	0.0518 (15)	0.0323 (12)	−0.0061 (14)	0.0031 (12)	−0.0078 (11)
C5	0.0519 (16)	0.0539 (15)	0.0313 (12)	0.0043 (13)	0.0066 (11)	−0.0011 (11)
C6	0.132 (4)	0.057 (2)	0.106 (3)	−0.017 (2)	0.020 (3)	−0.0030 (19)
C7	0.100 (3)	0.099 (3)	0.078 (2)	−0.024 (2)	0.031 (2)	0.015 (2)
C8	0.087 (3)	0.117 (3)	0.093 (3)	−0.050 (2)	−0.022 (2)	0.021 (2)
C9	0.076 (2)	0.087 (2)	0.0587 (19)	0.0257 (19)	−0.0049 (16)	0.0209 (16)
C10	0.058 (2)	0.128 (3)	0.0535 (18)	−0.026 (2)	−0.0131 (15)	−0.0047 (19)
C11	0.113 (3)	0.0591 (19)	0.0535 (19)	−0.0160 (18)	0.0105 (17)	−0.0199 (14)
C12	0.0612 (18)	0.0756 (19)	0.0480 (15)	0.0186 (16)	0.0116 (14)	−0.0002 (14)
C13	0.0559 (16)	0.0465 (14)	0.0312 (12)	−0.0038 (12)	−0.0022 (11)	−0.0059 (10)
C14	0.0590 (18)	0.0614 (17)	0.0336 (13)	0.0050 (14)	0.0056 (12)	−0.0072 (12)
C15	0.0519 (17)	0.083 (2)	0.0326 (13)	−0.0102 (15)	0.0078 (12)	−0.0002 (13)
C16	0.077 (2)	0.0521 (16)	0.0330 (13)	−0.0184 (15)	0.0013 (13)	0.0045 (12)
C17	0.0601 (17)	0.0506 (14)	0.0307 (12)	−0.0005 (13)	−0.0033 (11)	0.0040 (11)
C18	0.152 (4)	0.072 (2)	0.116 (3)	−0.049 (3)	−0.061 (3)	0.032 (2)
C19	0.080 (3)	0.135 (4)	0.101 (3)	−0.051 (3)	0.024 (2)	−0.045 (3)
C20	0.083 (2)	0.069 (2)	0.0545 (17)	−0.0225 (17)	−0.0116 (16)	−0.0048 (14)
C21	0.109 (3)	0.088 (2)	0.061 (2)	0.038 (2)	0.0071 (19)	−0.0238 (18)
C22	0.055 (2)	0.154 (4)	0.0571 (19)	−0.022 (2)	0.0145 (16)	0.000 (2)
C23	0.137 (4)	0.066 (2)	0.0508 (19)	−0.037 (2)	0.0057 (19)	0.0144 (15)
C24	0.088 (2)	0.078 (2)	0.0553 (18)	0.0249 (19)	−0.0211 (17)	0.0074 (15)
C25	0.0558 (17)	0.0467 (14)	0.0571 (17)	−0.0115 (13)	0.0026 (13)	−0.0053 (12)
C26	0.059 (2)	0.061 (2)	0.128 (3)	−0.0248 (17)	−0.0004 (19)	0.0077 (19)
C27	0.0600 (18)	0.0551 (16)	0.0606 (18)	0.0218 (14)	0.0013 (14)	−0.0018 (13)
C28	0.106 (3)	0.057 (2)	0.116 (3)	0.035 (2)	0.000 (2)	−0.0008 (19)
C29	0.0433 (14)	0.0387 (13)	0.0589 (16)	0.0031 (11)	−0.0022 (12)	−0.0003 (11)
C30	0.0507 (18)	0.0540 (17)	0.098 (3)	0.0115 (14)	−0.0022 (16)	0.0028 (16)

### Geometric parameters (Å, °)

**Table:**

Fe1—C13	2.104 (2)	C11—H11B	0.9600
Fe1—C14	2.111 (2)	C11—H11C	0.9600
Fe1—C15	2.126 (3)	C12—H12A	0.9600
Fe1—C16	2.136 (3)	C12—H12B	0.9600
Fe1—C17	2.123 (2)	C12—H12C	0.9600
Fe1—S1	2.2721 (7)	C13—C14	1.438 (4)
Fe1—S2	2.2765 (7)	C13—C17	1.445 (4)
Fe1—S3	2.2522 (7)	C14—C15	1.414 (4)
Fe2—C1	2.109 (2)	C14—C21	1.511 (4)
Fe2—C2	2.113 (2)	C15—C16	1.433 (4)
Fe2—C3	2.125 (2)	C15—C22	1.512 (4)
Fe2—C4	2.135 (2)	C16—C17	1.420 (4)
Fe2—C5	2.126 (2)	C16—C23	1.499 (4)
Fe2—S1	2.2659 (7)	C17—C24	1.497 (4)
Fe2—S2	2.2723 (7)	C18—H18A	0.9600
Fe2—S3	2.2545 (7)	C18—H18B	0.9600
Fe1—Fe2	2.7842 (5)	C18—H18C	0.9600
S1—C25	1.836 (3)	C19—H19A	0.9600
S2—C27	1.843 (3)	C19—H19B	0.9600
S3—C29	1.841 (2)	C19—H19C	0.9600
Si1—C18	1.858 (4)	C20—H20A	0.9600
Si1—C20	1.864 (3)	C20—H20B	0.9600
Si1—C19	1.867 (4)	C20—H20C	0.9600
Si1—C13	1.878 (3)	C21—H21A	0.9600
Si2—C8	1.859 (4)	C21—H21B	0.9600
Si2—C7	1.861 (3)	C21—H21C	0.9600
Si2—C6	1.865 (4)	C22—H22A	0.9600
Si2—C1	1.874 (3)	C22—H22B	0.9600
C1—C5	1.438 (4)	C22—H22C	0.9600
C1—C2	1.450 (4)	C23—H23A	0.9600
C2—C3	1.416 (4)	C23—H23B	0.9600
C2—C9	1.505 (4)	C23—H23C	0.9600
C3—C4	1.421 (4)	C24—H24A	0.9600
C3—C10	1.507 (4)	C24—H24B	0.9600
C4—C5	1.426 (4)	C24—H24C	0.9600
C4—C11	1.502 (4)	C25—C26	1.498 (4)
C5—C12	1.494 (4)	C25—H25A	0.9700
C6—H6A	0.9600	C25—H25B	0.9700
C6—H6B	0.9600	C26—H26A	0.9600
C6—H6C	0.9600	C26—H26B	0.9600
C7—H7A	0.9600	C26—H26C	0.9600
C7—H7B	0.9600	C27—C28	1.522 (4)
C7—H7C	0.9600	C27—H27A	0.9700
C8—H8A	0.9600	C27—H27B	0.9700
C8—H8B	0.9600	C28—H28A	0.9600
C8—H8C	0.9600	C28—H28B	0.9600
C9—H9A	0.9600	C28—H28C	0.9600
C9—H9B	0.9600	C29—C30	1.530 (4)
C9—H9C	0.9600	C29—H29A	0.9700
C10—H10A	0.9600	C29—H29B	0.9700
C10—H10B	0.9600	C30—H30A	0.9600
C10—H10C	0.9600	C30—H30B	0.9600
C11—H11A	0.9600	C30—H30C	0.9600
			
C13—Fe1—C14	39.90 (10)	H8A—C8—H8B	109.5
C13—Fe1—C17	39.98 (10)	Si2—C8—H8C	109.5
C14—Fe1—C17	65.67 (10)	H8A—C8—H8C	109.5
C13—Fe1—C15	66.85 (10)	H8B—C8—H8C	109.5
C14—Fe1—C15	38.98 (11)	C2—C9—H9A	109.5
C17—Fe1—C15	65.56 (11)	C2—C9—H9B	109.5
C13—Fe1—C16	66.94 (10)	H9A—C9—H9B	109.5
C14—Fe1—C16	65.66 (11)	C2—C9—H9C	109.5
C17—Fe1—C16	38.96 (10)	H9A—C9—H9C	109.5
C15—Fe1—C16	39.29 (12)	H9B—C9—H9C	109.5
C13—Fe1—S3	105.45 (7)	C3—C10—H10A	109.5
C14—Fe1—S3	144.15 (8)	C3—C10—H10B	109.5
C17—Fe1—S3	93.18 (8)	H10A—C10—H10B	109.5
C15—Fe1—S3	155.34 (9)	C3—C10—H10C	109.5
C16—Fe1—S3	116.12 (9)	H10A—C10—H10C	109.5
C13—Fe1—S1	162.12 (7)	H10B—C10—H10C	109.5
C14—Fe1—S1	123.23 (8)	C4—C11—H11A	109.5
C17—Fe1—S1	137.62 (7)	C4—C11—H11B	109.5
C15—Fe1—S1	95.73 (8)	H11A—C11—H11B	109.5
C16—Fe1—S1	102.55 (8)	C4—C11—H11C	109.5
S3—Fe1—S1	92.12 (3)	H11A—C11—H11C	109.5
C13—Fe1—S2	102.76 (7)	H11B—C11—H11C	109.5
C14—Fe1—S2	94.83 (8)	C5—C12—H12A	109.5
C17—Fe1—S2	140.11 (7)	C5—C12—H12B	109.5
C15—Fe1—S2	120.88 (9)	H12A—C12—H12B	109.5
C16—Fe1—S2	159.45 (9)	C5—C12—H12C	109.5
S3—Fe1—S2	83.31 (2)	H12A—C12—H12C	109.5
S1—Fe1—S2	82.28 (2)	H12B—C12—H12C	109.5
C13—Fe1—Fe2	143.59 (7)	C14—C13—C17	105.5 (2)
C14—Fe1—Fe2	145.80 (8)	C14—C13—Si1	128.1 (2)
C17—Fe1—Fe2	144.60 (7)	C17—C13—Si1	126.1 (2)
C15—Fe1—Fe2	145.90 (8)	C14—C13—Fe1	70.31 (14)
C16—Fe1—Fe2	145.06 (8)	C17—C13—Fe1	70.73 (13)
S3—Fe1—Fe2	51.882 (17)	Si1—C13—Fe1	128.11 (13)
S1—Fe1—Fe2	52.053 (18)	C15—C14—C13	109.6 (2)
S2—Fe1—Fe2	52.191 (17)	C15—C14—C21	124.4 (3)
C1—Fe2—C2	40.17 (10)	C13—C14—C21	125.6 (3)
C1—Fe2—C3	66.79 (11)	C15—C14—Fe1	71.08 (15)
C2—Fe2—C3	39.02 (11)	C13—C14—Fe1	69.78 (13)
C1—Fe2—C5	39.70 (10)	C21—C14—Fe1	131.4 (2)
C2—Fe2—C5	65.74 (10)	C14—C15—C16	108.0 (2)
C3—Fe2—C5	65.35 (11)	C14—C15—C22	126.3 (3)
C1—Fe2—C4	66.83 (10)	C16—C15—C22	125.6 (3)
C2—Fe2—C4	65.66 (11)	C14—C15—Fe1	69.94 (14)
C3—Fe2—C4	38.95 (11)	C16—C15—Fe1	70.72 (15)
C5—Fe2—C4	39.10 (10)	C22—C15—Fe1	127.8 (2)
C1—Fe2—S3	108.97 (7)	C17—C16—C15	107.5 (2)
C2—Fe2—S3	148.93 (8)	C17—C16—C23	125.6 (3)
C3—Fe2—S3	149.72 (9)	C15—C16—C23	126.2 (3)
C5—Fe2—S3	91.91 (7)	C17—C16—Fe1	70.06 (14)
C4—Fe2—S3	110.91 (8)	C15—C16—Fe1	69.99 (15)
C1—Fe2—S1	158.81 (7)	C23—C16—Fe1	133.0 (2)
C2—Fe2—S1	118.70 (8)	C16—C17—C13	109.4 (2)
C3—Fe2—S1	94.88 (8)	C16—C17—C24	124.3 (3)
C5—Fe2—S1	143.01 (8)	C13—C17—C24	126.1 (3)
C4—Fe2—S1	106.08 (8)	C16—C17—Fe1	70.99 (15)
S3—Fe2—S1	92.22 (3)	C13—C17—Fe1	69.28 (13)
C1—Fe2—S2	99.65 (7)	C24—C17—Fe1	130.47 (19)
C2—Fe2—S2	96.94 (8)	Si1—C18—H18A	109.5
C3—Fe2—S2	126.74 (9)	Si1—C18—H18B	109.5
C5—Fe2—S2	134.48 (8)	H18A—C18—H18B	109.5
C4—Fe2—S2	162.58 (8)	Si1—C18—H18C	109.5
S3—Fe2—S2	83.35 (2)	H18A—C18—H18C	109.5
S1—Fe2—S2	82.51 (2)	H18B—C18—H18C	109.5
C1—Fe2—Fe1	143.52 (7)	Si1—C19—H19A	109.5
C2—Fe2—Fe1	146.54 (8)	Si1—C19—H19B	109.5
C3—Fe2—Fe1	146.66 (8)	H19A—C19—H19B	109.5
C5—Fe2—Fe1	143.71 (7)	Si1—C19—H19C	109.5
C4—Fe2—Fe1	144.59 (8)	H19A—C19—H19C	109.5
S3—Fe2—Fe1	51.807 (18)	H19B—C19—H19C	109.5
S1—Fe2—Fe1	52.256 (17)	Si1—C20—H20A	109.5
S2—Fe2—Fe1	52.330 (18)	Si1—C20—H20B	109.5
C25—S1—Fe2	112.23 (9)	H20A—C20—H20B	109.5
C25—S1—Fe1	113.25 (10)	Si1—C20—H20C	109.5
Fe2—S1—Fe1	75.69 (2)	H20A—C20—H20C	109.5
C27—S2—Fe2	109.33 (10)	H20B—C20—H20C	109.5
C27—S2—Fe1	108.55 (10)	C14—C21—H21A	109.5
Fe2—S2—Fe1	75.48 (2)	C14—C21—H21B	109.5
C29—S3—Fe1	110.79 (9)	H21A—C21—H21B	109.5
C29—S3—Fe2	110.84 (9)	C14—C21—H21C	109.5
Fe1—S3—Fe2	76.31 (2)	H21A—C21—H21C	109.5
C18—Si1—C20	107.53 (17)	H21B—C21—H21C	109.5
C18—Si1—C19	107.3 (2)	C15—C22—H22A	109.5
C20—Si1—C19	108.69 (18)	C15—C22—H22B	109.5
C18—Si1—C13	112.45 (16)	H22A—C22—H22B	109.5
C20—Si1—C13	107.90 (12)	C15—C22—H22C	109.5
C19—Si1—C13	112.76 (14)	H22A—C22—H22C	109.5
C8—Si2—C7	110.3 (2)	H22B—C22—H22C	109.5
C8—Si2—C6	106.3 (2)	C16—C23—H23A	109.5
C7—Si2—C6	106.96 (18)	C16—C23—H23B	109.5
C8—Si2—C1	112.19 (14)	H23A—C23—H23B	109.5
C7—Si2—C1	107.36 (15)	C16—C23—H23C	109.5
C6—Si2—C1	113.59 (16)	H23A—C23—H23C	109.5
C5—C1—C2	105.6 (2)	H23B—C23—H23C	109.5
C5—C1—Si2	124.7 (2)	C17—C24—H24A	109.5
C2—C1—Si2	129.3 (2)	C17—C24—H24B	109.5
C5—C1—Fe2	70.80 (14)	H24A—C24—H24B	109.5
C2—C1—Fe2	70.08 (14)	C17—C24—H24C	109.5
Si2—C1—Fe2	129.16 (13)	H24A—C24—H24C	109.5
C3—C2—C1	108.8 (2)	H24B—C24—H24C	109.5
C3—C2—C9	123.8 (3)	C26—C25—S1	111.5 (2)
C1—C2—C9	126.9 (3)	C26—C25—H25A	109.3
C3—C2—Fe2	70.95 (15)	S1—C25—H25A	109.3
C1—C2—Fe2	69.75 (13)	C26—C25—H25B	109.3
C9—C2—Fe2	131.4 (2)	S1—C25—H25B	109.3
C2—C3—C4	108.6 (2)	H25A—C25—H25B	108.0
C2—C3—C10	125.5 (3)	C25—C26—H26A	109.5
C4—C3—C10	125.8 (3)	C25—C26—H26B	109.5
C2—C3—Fe2	70.03 (14)	H26A—C26—H26B	109.5
C4—C3—Fe2	70.88 (14)	C25—C26—H26C	109.5
C10—C3—Fe2	127.51 (19)	H26A—C26—H26C	109.5
C3—C4—C5	107.5 (2)	H26B—C26—H26C	109.5
C3—C4—C11	125.3 (3)	C28—C27—S2	111.5 (3)
C5—C4—C11	126.2 (3)	C28—C27—H27A	109.3
C3—C4—Fe2	70.16 (14)	S2—C27—H27A	109.3
C5—C4—Fe2	70.12 (14)	C28—C27—H27B	109.3
C11—C4—Fe2	134.0 (2)	S2—C27—H27B	109.3
C4—C5—C1	109.4 (2)	H27A—C27—H27B	108.0
C4—C5—C12	124.8 (3)	C27—C28—H28A	109.5
C1—C5—C12	125.7 (3)	C27—C28—H28B	109.5
C4—C5—Fe2	70.77 (14)	H28A—C28—H28B	109.5
C1—C5—Fe2	69.50 (13)	C27—C28—H28C	109.5
C12—C5—Fe2	128.69 (18)	H28A—C28—H28C	109.5
Si2—C6—H6A	109.5	H28B—C28—H28C	109.5
Si2—C6—H6B	109.5	C30—C29—S3	111.25 (19)
H6A—C6—H6B	109.5	C30—C29—H29A	109.4
Si2—C6—H6C	109.5	S3—C29—H29A	109.4
H6A—C6—H6C	109.5	C30—C29—H29B	109.4
H6B—C6—H6C	109.5	S3—C29—H29B	109.4
Si2—C7—H7A	109.5	H29A—C29—H29B	108.0
Si2—C7—H7B	109.5	C29—C30—H30A	109.5
H7A—C7—H7B	109.5	C29—C30—H30B	109.5
Si2—C7—H7C	109.5	H30A—C30—H30B	109.5
H7A—C7—H7C	109.5	C29—C30—H30C	109.5
H7B—C7—H7C	109.5	H30A—C30—H30C	109.5
Si2—C8—H8A	109.5	H30B—C30—H30C	109.5
Si2—C8—H8B	109.5		
